# Supplementary material for: Transcriptome and DNA Methylome Analysis of Two Contrasting Rice Genotypes under Salt Stress during Germination
Source: Int J Mol Sci. 2023 Feb 16;24(4):3978. doi: 10.3390/ijms24043978 (PMC9965394; doi:10.3390/ijms24043978)
Supplement: Supplementary file 1 [file ijms-24-03978-s001.zip › ijms-2106032-supplementary.pdf]

Supplemental materials

Transcriptome and DNA Methylome Analysis of Two Contrasting Rice Genotypes under Salt Stress during Germination

Yongqiang Li and Dianjing Guo *

State Key Laboratory of Agrobiotechnology, School of Life Sciences, The Chinese University of Hong Kong, Hong Kong 999077, China
* Correspondence: djguo@cuhk.edu.hk

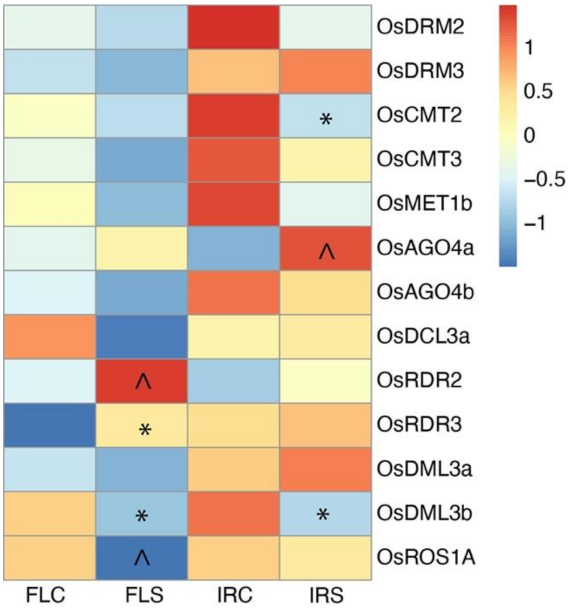

Figure S1. The expression heatmap of DNA methylase and demethylases, and RdDM pathway components. *: 2-fold changes. ^: 1.5-fold changes. Z-score. FLC: FL478 control. FLS: FL478 salt. IRC: IR29 control. IRS: IR29 salt.

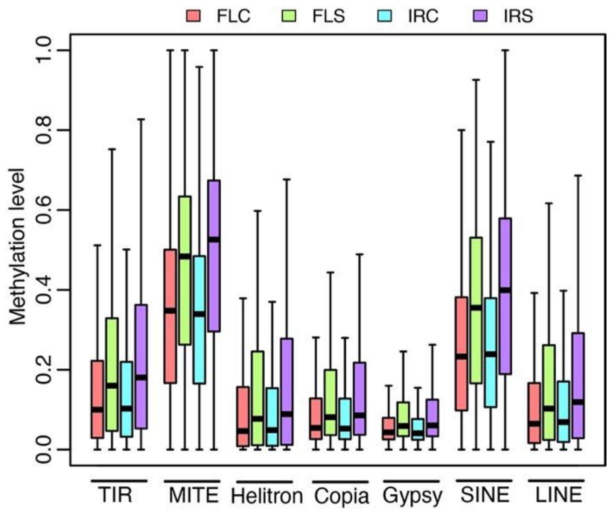

Figure S2. The CHH methylation level of all kinds TEs. Pink FLC: FL478 control. Green FLS: FL478 salt. Lightblue IRC: IR29 control. Purple IRS: IR29 salt.

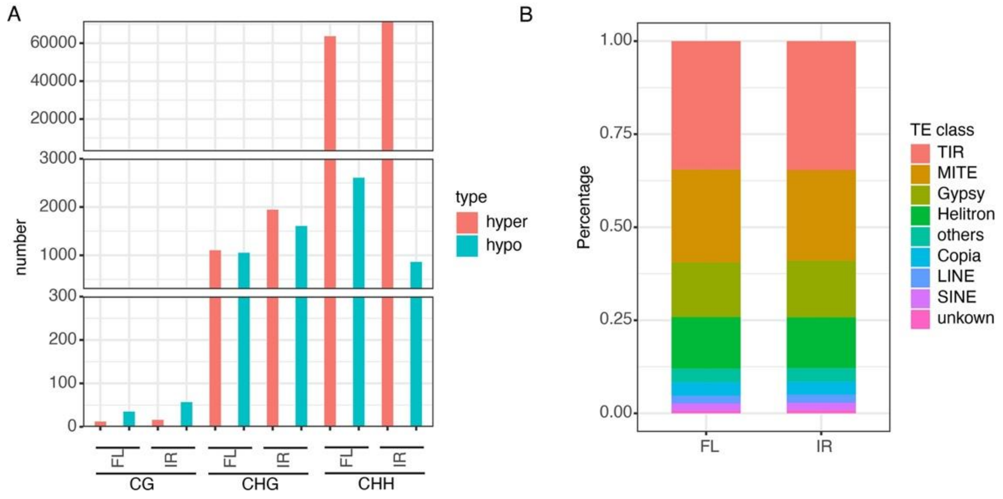

Figure S3. The summary of DMR and CHH hyper DMR associated TE classification. (A) The number of hyper and hypo DMRs in three contexts. (B) The percentage of hyper CHH DMRs associated TEs. FL: FL478. IR: IR29.

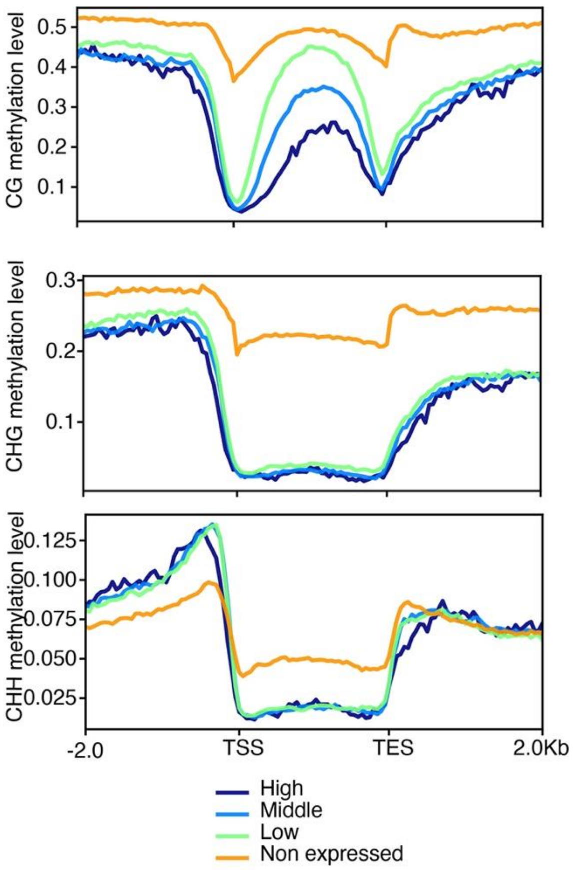

Figure S4. The relationship between gene expression and DNA methylation. Top: CG methylation level and gene expression. Middle: CHG methylation level and gene expression. Bottom: CHH methylation level and gene expression. Genes were classified into four classes based on expression value. Blue: genes with high expression values. Lightblue: genes with middle expression values. Green: genes with low expression values. Orange: non-expressed genes.
